# Undercarboxylated osteocalcin reverts insulin resistance induced by endoplasmic reticulum stress in human umbilical vein endothelial cells

**DOI:** 10.1038/s41598-017-00163-2

**Published:** 2017-03-03

**Authors:** Qinyue Guo, Huixia Li, Lin Xu, Shufang Wu, Hongzhi Sun, Bo Zhou

**Affiliations:** 1 Department of Respiratory and Critical Care Medicine, the First Affiliated Hospital of Xi’an Jiaotong University, 277 Yanta West Road, Xi’an, Shaanxi 710061 China; 2 Key Laboratory of Environment and Genes Related to Diseases, Medical School of Xi’an Jiaotong University, Xi’an, Shaanxi 710061 China; 3 Department of Endocrinology, the Affiliated Guangren Hospital of Xi’an Jiaotong University, Xi’an, Shaanxi 710004 China; 4 Center for Translational Medicine, the First Affiliated Hospital of Xi’an Jiaotong University, 277 Yanta West Street, Xi’an, Shaanxi 710061 China

## Abstract

Osteocalcin has been considered to be an important regulator of energy metabolism in type 2 diabetes mellitus (T2DM). However, the mechanism underlying the involvement of uncarboxylated osteocalcin in the vascular complications of T2DM is not fully understood. In the present study, we analyzed the potential correlations between uncarboxylated osteocalcin and macro- or microangiopathic complications in subjects with T2DM and tested the impact of uncarboxylated osteocalcin on insulin resistance in human umbilical vein endothelial cells (HUVECs). The results showed that the serum levels of uncarboxylated osteocalcin were lower in subjects with vascular complications of T2DM. Univariate correlation analyses revealed negative correlations between uncarboxylated osteocalcin and waist-to-hip ratio, HbA1c, and HOMA-IR. In *in vitro* experiments, insulin resistance was induced by applying tunicamycin to HUVECs. Uncarboxylated osteocalcin not only markedly reduced the phosphorylations of PERK and eIF2α, but also elevated the phosphorylations of IRS-1 and Akt, resulting in improvement of insulin signal transduction via PI3K/Akt/NF-κB signaling in HUVECs. Therefore, there is a possible relationship between uncarboxylated osteocalcin and the vascular complications of T2DM. Uncarboxylated osteocalcin partially improves insulin signal transduction via PI3K/Akt/NF-κB signaling in tunicamycin-induced HUVECs, suggesting osteocalcin as a potential treatment for the vascular complications of T2DM.

## Introduction

Osteocalcin (OCN) is an osteoblast-specific protein that exhibits several features of a hormone^1^. Besides its functions in bone mineralization, OCN has recently emerged as a key regulator of glycolipid metabolism, particularly in its uncarboxylated form, including favoring pancreatic β-cell proliferation and enhancing insulin sensitivity^2, 3^. Recent studies found that mice lacking the osteoblast-specific tyrosine phosphatase Esp showed a higher circulating level of uncarboxylated osteocalcin (ucOCN) and greater protection from diabetes. This insulin-sensitive phenotype was abrogated by deleting a single allele of OCN, and OCN-knockout mice finally became resistant to insulin and hyperglycemic with age^4^. Moreover, the intermittent or continuous delivery of OCN promoted insulin sensitivity and reduced the severity of obesity in mice consuming a high-fat diet^5, 6^. Although the role of OCN (especially ucOCN) in energy metabolism has recently been identified, the clinical function of ucOCN remains to be elucidated.

Several clinical investigations have found significant inverse associations of total osteocalcin (totOCN) and ucOCN with insulin resistance, blood glucose, adiposity, and triglycerides levels^7–11^. Serum OCN concentrations were recently found to be negatively correlated with carotid atherosclerosis and peripheral vascular disease, and reduced OCN levels were involved in all-cause mortality and cardiovascular disease (CVD)-related deaths in older men^12^, adding more evidence for the correlation between OCN and CVD. There is an increasing prevalence of type 2 diabetes mellitus (T2DM) being associated with premature atherosclerosis and microvascular complications, but few studies have investigated the links between ucOCN and macro- or microangiopathic complications of T2DM.

Injecting OCN inhibits ER stress and improves insulin signaling in the vascular tissue of obese mice, which possibly implicates OCN, as a bone-derived hormone, in the development of CVD *in vivo* ^13, 14^. However, the intracellular mechanisms responsible for OCN-mediated effects on insulin resistance in human umbilical vein endothelial cells (HUVECs) remain elusive. There are recent results showing that NF-κB—as a important signaling molecular in cardiovascular system—is associated with a reduced responsiveness to insulin, and exerts a regulatory role via PI3K/Akt/NF-κB signaling^15^. In light of these observations, we postulated that ucOCN plays a critical role in the vascular complications of T2DM via PI3K/Akt/NF-κB signaling.

In the present study, we evaluated the correlations between ucOCN and macro- or microangiopathic complications in the progression of T2DM. We provide evidence that ucOCN reverses ER stress and restores insulin signaling via PI3K/Akt/NF-κB signaling in HUVECs, adding credence to the emerging notion that ucOCN could be used in treatments for the vascular complications of T2DM.

## Materials and Methods

### Subjects

Informed consent was obtained from all study participants in advance, and all procedures were performed in accordance with the guidelines in the Declaration of Helsinki, and were approved by the ethics committee, Xi’an Jiao Tong University. We included a cohort with a total number of 98 male individuals in our study of serum uncarboxylated and total osteocalcin concentration from 2010 to 2012. Study patients were selected when diagnosed with T2DM: one of FPG ≥ 7.0 mmol/l, G(120) that is the plasma glucose level at 120 min during 75 g-OGTT ≥ 11.1 mmol/l and HbA1c ≥ 6.5% was fulfilled. All diabetic patients were checked in the clinic for following vascular complications: (1) Ischemic heart disease was clinically assessed (history of a cardiovascular event and/or presence of angina) and in addition, a 12-lead resting electrocardiogram was recorded supine (Mac PC Electrocardiograph, Marquette Electronics, Los Angeles, CA) and evaluated by a cardiologist. The presence of any of the following findings was considered suggestive of coronary heart disease: T-wave inversion, ST-segment depression, and Q waves. (2) Patients with indicative signs or symptoms of cerebrovascular disease were evaluated with a CNS computerized tomography. The final diagnosis was reviewed by a neurologist. (3) Peripheral vascular disease was clinically defined by the presence of intermittent claudication, absent or weakened peripheral pulses, or both (no amputations were detected in our population). (4) Retinopathy was documented by standard fundus eye examination and diagnosed on the presence of microaneurysms, venous dilatation, cotton-wool spots, neovascularization, or hemorrhages. (5) Clinical neuropathy was defined by an abnormal neurologic examination, consistent with the presence of peripheral sensorimotor neuropathy. (6) Nephropathy was defined by the presence of urinary albumin excretion of >30 mg 24-h and/or high plasma creatinine levels. Prediabetes (PDM) includes impaired fasting glucose (IFG: 6.1 mmol/l ≤ FPG < 7.0 mmol/l) or impaired glucose tolerance (IGT: 7.8 mmol/l ≤ G(120) < 11.1 mmol/l). Normal glucose tolerance (NGT) indicates none of the above criteria. All participants gave written informed consent before taking part in.

### Anthropometric and biochemical measurements

All baseline blood samples were collected between 8:00 and 10:00 A.M. after an overnight fast. Blood samples were stored at −80 °C prior to analysis. A complete physical examination including the measurement of height, weight, waist circumference and hip circumference was performed on each subject. Body mass index (BMI) was calculated as weight/height^2^ (kg/m^2^). Waist circumference was measured at the midpoint between the lower ribs and iliaccrest; hip circumference was measured over the buttocks. Waist hip rate (WHR) was calculated as waist circumference/hip circumference. Plasma glucose was determined using glucose oxidase method. Serum insulin was assayed via radio-immunoassay (Linco Research, St Charles, MO, USA). Glycated haemoglobin A1c (HbA1c) values were measured by high-performance liquid chromatography (Bio-Rad Laboratories, Hercules, CA, USA). Serum lipid profiles, including total cholesterol (TC), triglyceride (TG), high-density lipoprotein cholesterol (HDL-c) and low density lipoprotein cholesterol (LDL-c) were determined by enzymatic procedures on an autoanalyser (Hitachi 7600-020; Hitachi, Tokyo, Japan). Homeostasis model assessment index for insulin resistance (HOMA-IR) were used to estimate insulin sensitivity.

### Uncarboxylated and total osteocalcin quantification

Peripheral serum was harvested from peripheral blood after centrifugation of red blood cells. Peripheral serum were subject to ELISA using standard kits (R&D Systems, Inc., Minneapolis, MN, USA) for uncarboxylated and total osteocalcin concentration.

### Purification of recombinant uncarboxylated osteocalcin

Bacterially produced mouse recombinant uncarboxylated osteocalcin was purified as described previously^5^. In brief, glutathione S-transferase-osteocalcin fusion protein was produced and purified on glutathione-sepharose in accordance with the standard procedure. Thrombin was then used to cleave out osteocalcin from the GST moiety, and the high purity of the osteocalcin preparation (>95%) was confirmed by Tris-tricine SDS-PAGE staining. An osteocalcin RIA kit (Immunotope, Doylestown, Pennsylvania) was used to measure the concentration of the recombinant osteocalcin protein.

### Cell culture and treatment

HUVECs were purchased from the American Type Culture Collection (ATCC, Manassas, VA). HUVECs were cultured in DMEM/F-12 (HyClone, Thermo Fisher Scientific Inc. Logan, UT) containing 10% fetal bovine serum (FBS) (HyClone, Thermo Fisher Scientific Inc. Logan, UT). Insulin resistance was induced by applying pretreatment with 5 μg/ml tunicamycin for 4 h. The effects of uncarboxylated osteocalcin were determined by treating cells with 5 ng/ml of uncarboxylated osteocalcin for 4 h. Insulin signaling in the cells was stimulated by applying 10 nM insulin for 10 min. The medium was replaced with fresh medium prior to each experiment.

### Western blot

Tissues and cells under various treatments were lysed in lysis buffer containing 25 mM Tris HCl (pH 6.8), 2% SDS, 6% glycerol, 1% 2-mercaptoethanol, 2 mM phenylmethylsulfonyl fluoride, 0.2% bromophenol blue, and a protease inhibitor cocktail for 20 min and boiled for another 5 min. The protein concentration was determined by Coomassie brilliant blue protein assay. Equal amounts of total proteins (20 μg) underwent 15% SDS-PAGE and were electroblotted onto polyvinylidene difluoride membrane. The membrane was blocked with 5% (w/v) nonfat dry milk or albumin from bovine serum in PBS–polysorbate 20 (PBST; 0.1%) for 1 h and incubated with primary antibodies at 4 °C overnight. After three times washing in PBST, the membrane was incubated with appropriate horseradish peroxidase–conjugated secondary antibodies for 1 h at room temperature. Then the membrane was developed using the enhanced chemiluminescence system. The relative quantity of proteins was analyzed using Quantity One software (Bio-Rad, Hercules, CA).

### Immunoprecipitation

200 μg of cytoplasmic lysate were incubated for 2 h at 4 °C with the corresponding antibodies coupled to 20 μl of packed protein A + G sepharose beads (Beyotime, Jiangsu, China). Immunocomplexes were resolved by means of SDS-PAGE and immuno-blotted with the indicated antibodies.

### PI3K assay

PI3K activities were determined by an *in vitro* kinase assay (SuperArray Bioscience Corporation, Frederick, MD, USA), according to manufacturer’s instructions. HUVECs were seeded into 96-well plates and incubated with 5 ng/ml of uncarboxylated osteocalcin for 4 h. Cells were then fixed with 4% formaldehyde for 20 min at room temperature to preserve phosphorylation. The relative extent of target protein phosphorylation is determined by normalizing absorbance reading of the phospho-protein specific antibody to the pan-protein specific antibody for the same experimental condition. Data were expressed as percentage of control.

### NF-κB p65 transcription factor assay

NF-κB p65 transcription factor activity was determined using an ELISA kit (Cayman Chemical, Ann Arbor, MI, USA). According to the manufacturer’s instructions, nuclei were extracted from these cells treated 5 ng/ml of uncarboxylated osteocalcin for 4 h, and/or the following specific protein kinase inhibitors: wortmannin (a PI3K inhibitor); Akti-1/2 (an AKT inhibitor) and U0126 (a MAPK inhibitor). Data are expressed as absorbance at 450 nm/mg protein.

### Gene silencing

HUVECs were transfected with a siRNA targeted for human NF-κB-p65 (Santa Cruz Biotechnology, catalogue number sc-29410) using Lipofectamine 2000 (Invitrogen, Carlsbad, CA). siRNA consisting of a scrambled sequence of similar length was similarly transfected as control siRNA. One day before transfection, the cells were plated in 500 μl of growth medium without antibiotics so as to achieve 30% to 50% confluence at the time of transfection. The transfected cells were then cultured for 72 h in DMEM containing 10% fetal calf serum. The knockdown efficiency was determined by Western blot to measure the expression levels of NF-κB-p65 proteins in transfected cells.

### Glucose uptake

After transfer of HUVECs to medium without glucose, HUVECs were incubated with 10 nmol/l insulin for 15 min, when glucose transport was determined as uptake of 50 mmol/l (10 mCi/ml) 2-deoxy-D-[1-3H] glucose, and then incubated 30 min. Uptake was linear for at least 30 min.

### Statistical analyses

Data are shown as means ± SE unless stated otherwise. Before statistical analysis, non-normally distributed parameters were logarithmically transformed to approximate a normal distribution. Statistical analysis was performed using unpaired, two-tailed Student t-test or ANOVA followed by *post hoc* tests. Multiple logistic regression analysis was performed to identify independent predictors for the vascular complications of T2DM. Univariate correlation analyses were used to assess the correlations between serum osteocalcin and other continuous parameters. Statistical analyses were performed using SPSS 17.0 software. Differences were considered significant when the *P* value was less than 0.05.

## Results

### Basic characteristics of the study subjects

The anthropometric and metabolic characteristics of the 98 individuals included in the cross-sectional study are summarized in Table 1. Subjects diagnosed with T2DM with or without vascular complications exhibited higher FPG, FINS, HbA1c, TC, TG, LDL-c and HOMA-IR compared with NGT. Additionally, eGFR and adiponectin levels were decreased in the group of T2DM with vascular complications.

**Table 1:** Anthropometric and metabolic characteristics of the study groups.

	NGT	PDM	T2DM Without vascular complications	T2DM With vascular complications
n	22	18	25	33
Age (years)	48.2 ± 7.3	49.6 ± 9.2	50.7 ± 8.3	51.8 ± 6.6
BMI (kg/m^2^)	23.5 ± 2.1	26.1 ± 1.7^#^	26.5 ± 3.3*	27.0 ± 2.6*
WHR	0.82 ± 0.03	0.91 ± 0.06^#^	0.88 ± 0.05	0.90 ± 0.07*
FPG (mmol/l)	5.14 ± 0.24	5.38 ± 0.51	7.25 ± 0.81*	7.83 ± 0.62*
FINS (mU/l)	9.82 ± 1.32	12.04 ± 1.74	20.86 ± 2.75*	25.96 ± 2.95*
HbA1c (%)	5.21 ± 0.27	5.68 ± 0.39	6.08 ± 1.14*	6.86 ± 0.98*
TC (mmol/l)	4.60 ± 0.26	4.88 ± 0.75	5.09 ± 0.87*	5.71 ± 0.93*
TG (mmol/l)	1.21 ± 0.36	1.45 ± 0.45	2.13 ± 0.52*	2.65 ± 0.90*
HDL-c (mmol/l)	1.42 ± 0.82	1.35 ± 0.41	1.12 ± 0.12*	1.01 ± 0.23*
LDL-c (mmol/l)	3.12 ± 0.28	3.36 ± 0.34	3.58 ± 0.49	3.62 ± 0.52*
eGFR (ml/min/1.73 m^2^)	126.92 ± 10.61	121.24 ± 11.38	115.71 ± 8.16	58.75 ± 7.83*
Adiponectin (mg/l)	12.28 ± 2.23	9.14 ± 0.72^#^	7.09 ± 0.55*	4.83 ± 0.37*
HOMA-IR	2.15 ± 0.31	2.87 ± 0.55^#^	7.51 ± 0.86*	7.85 ± 2.29*

BMI, body mass index; eGFR, estimated glomerular filtration rate; FINS, fasting plasma insulin; FPG, fasting plasma glucose; HbA1c, glycated haemoglobin A1c; HDL-c, high-density lipoprotein cholesterol; HOMA-IR, homeostasis model assessment index of insulin resistance; LDL-c, low-density lipoprotein cholesterol; NGT, normal glucose tolerance; PDM, prediabetes; TC, total cholesterol; TG, triglyceride; WHR, waist-to-hip ratio. Data are means ± SD or median (interquartile range).

*P < 0.05 for patients with type 2 diabetes versus normal glucose tolerant group.

^#^ *P* < 0.05 obesity of prediabetes group versus normal glucose tolerant group.

### Serum ucOCN and totOCN concentrations in the subjects

**Figure 1:**
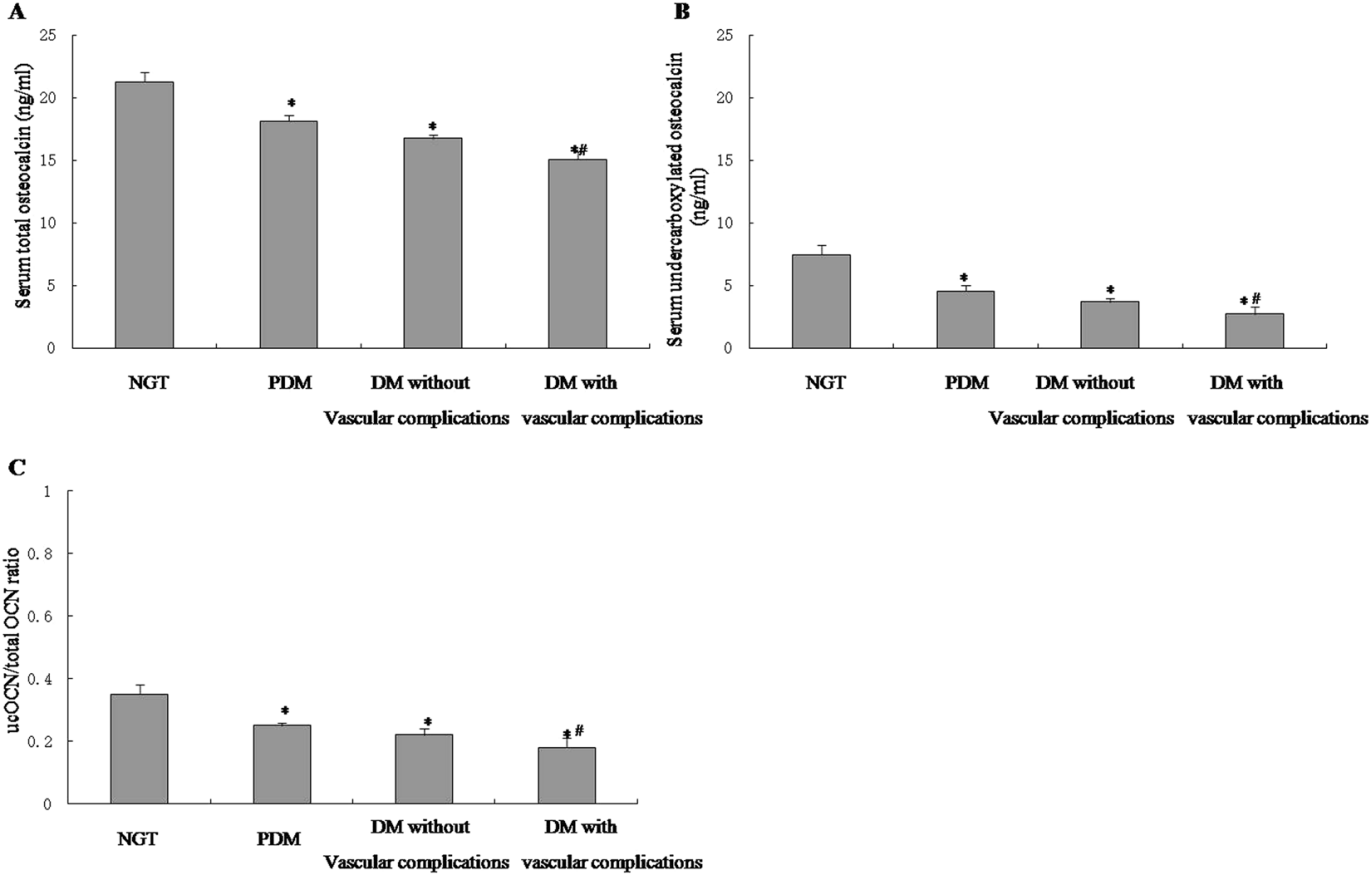
Serum ucOCN and totOCN concentrations in the subjects. Uncarboxylated and total osteocalcin levels were determined by ELISA. (**A**) Serum osteocalcin. (**B**) Serum uncarboxylated osteocalcin. (**C**) ucOCN/total OCN ratio. The data was expressed as mean ± SEM in each bar graph. *P < 0.05 (PDM, T2DM with or without vascular complications vs. NGT). ^#^P < 0.05 (T2DM with vascular complications vs. T2DM without vascular complications).

To determine the role of ucOCN and totOCN in T2DM with vascular complications, we used ELISA to measure the serum ucOCN and totOCN concentrations. As shown in Fig. 1A–C, the serum ucOCN and totOCN concentrations and the ucOCN/totOCN ratio were significantly lower in PDM and T2DM than in NGT. When T2DM subjects were further divided into different groups according to the vascular complications of T2DM, the serum ucOCN and totOCN concentrations and the ucOCN/totOCN ratio were significantly lower in T2DM with vascular complications than in T2DM without vascular complications (Fig. 1A–C).

Multiple logistic regression analysis performed using the presence of the vascular complications of T2DM as a dependent variable revealed that serum ucOCN and totOCN, BMI, FPG, HbA1c, and HOMA-IR were independent predictors for the development of the vascular complications (Table 2). Meanwhile, after adjusting for age and BMI, univariate correlation analyses revealed negative correlations between ucOCN and WHR, FPG, FINS, HbA1c, and HOMA-IR (Table 3). A positive correlation was also observed between ucOCN and eGFR (Table 3). Correlations with serum totOCN concentrations were generally in the same direction as those with ucOCN (Table 3).

**Table 2:** Multiple logistic regression analysis showing factors independently associated with vascular complications of type 2 diabetes.

Parameters	OR	95%CI	*P*
ucOCN	0.072	0.006–0.682	0.025
Total OCN	0.065	0.005–0.642	0.021
BMI	1.271	1.202–1.462	0.001
FPG	1.652	1.158–2.359	0.004
HbA1c	2.205	1.235–3.756	0.007
HOMA-IR	8.348	1.352–52.156	0.023

BMI, body mass index; FPG, fasting plasma glucose; HbA1c, glycated haemoglobin A1c; HOMA-IR, homeostasis model assessment index of insulin resistance; ucOCN, uncarboxylated osteocalcin.

**Table 3:** Correlation of serum total and uncarboxylated osteocalcin levels with anthropometric parameters and biochemical indexes.

	ucOCN (adjust for age and BMI)		Total OCN (adjust for age and BMI)	
	r	*P*	r	*P*
WHR	−0.275	<0.001	−0.268	<0.001
FPG (mmol/l)	−0.312	<0.001	−0.295	<0.001
FINS (mU/l)	−0.225	0.004	−0.182	0.017
HbA1c (%)	−0.175	0.024	−0.185	0.015
TC (mmol/l)	−0.115	0.125	−0.086	0.278
TG (mmol/l)	−0.103	0.199	−0.108	0.161
HDL-c (mmol/l)	0.084	0.280	0.138	0.070
LDL-c (mmol/l)	−0.122	0.116	0.135	0.089
eGFR (ml/min/1.73 m^2^)	0.172	0.022	0.164	0.035
HOMA-IR	−0.194	0.012	−0.202	0.009

BMI, body mass index; eGFR, estimated glomerular filtration rate; FINS, fasting plasma insulin; FPG, fasting plasma glucose; HbA1c, glycated haemoglobin A1c; HDL-c, high-density lipoprotein cholesterol; HOMA-IR, homeostasis model assessment index of insulin resistance; LDL-c, low-density lipoprotein cholesterol; TC, total cholesterol; TG, triglyceride; WHR, waist-to-hip ratio.

### Effects of ucOCN on tunicamycin-induced ER stress and impaired insulin signaling in HUVECs

**Figure 2:**
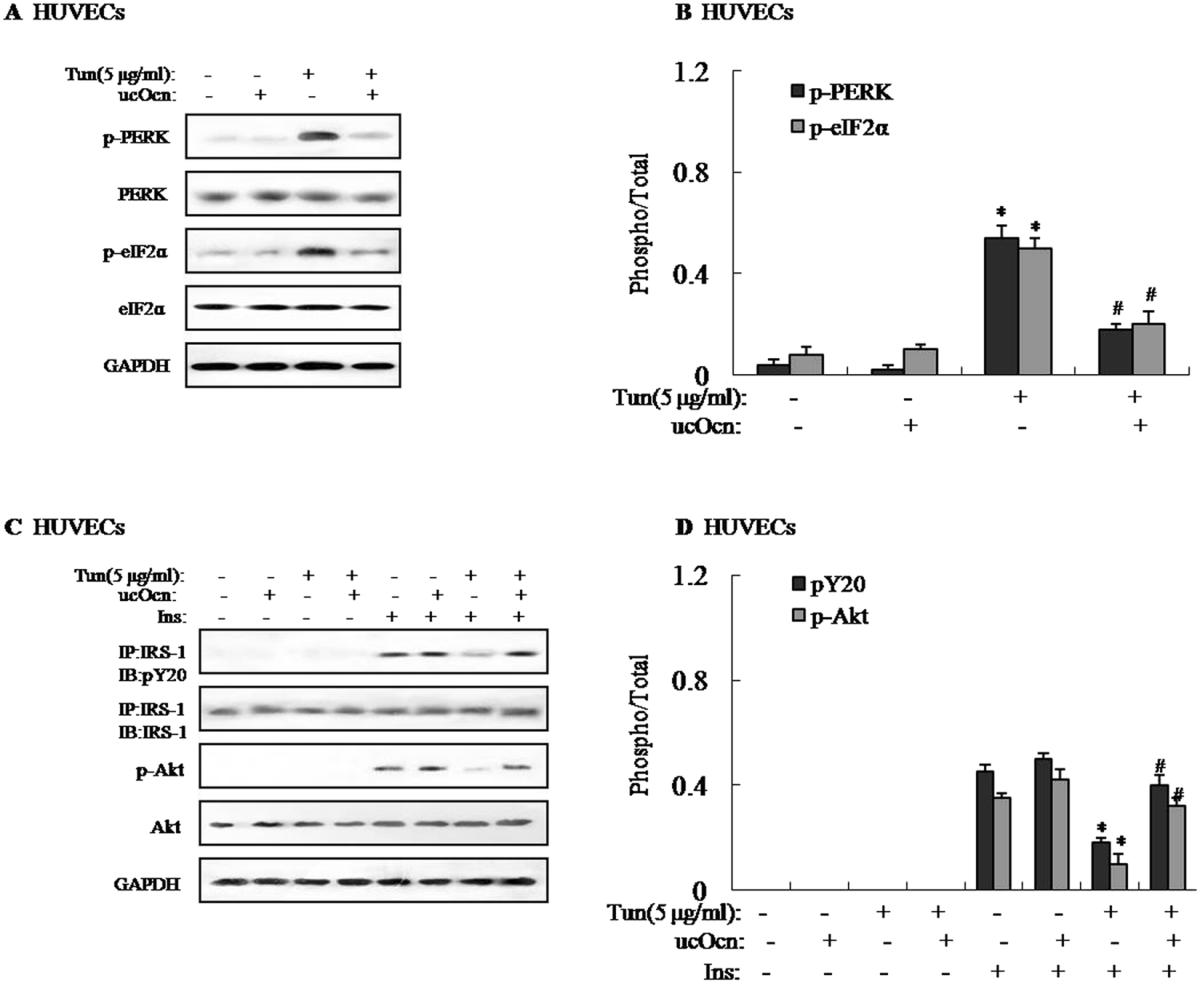
Effects of ucOCN on tunicamycin-induced ER stress and impaired insulin signaling in HUVECs. Tunicamycin (Tun) was used to induce insulin resistance. To determine the effects of uncarboxylated osteocalcin, HUVECs were treated with 5 ng/ml of uncarboxylated osteocalcin for 4 h. For insulin signaling, cells were stimulated with 10 nM of insulin for 10 min. The relative quantity of proteins was analyzed using Quantity One software. (**A**) Phosphorylation of PERK and eIF2α in HUVECs. (**B**) Densitometric analyses of PERK and eIF2α in HUVECs. (**C**) IRS-1 tyrosine phosphorylation and Akt Ser-473 phosphorylation in HUVECs. (**D**) Densitometric analyses of IRS-1 tyrosine phosphorylation and Akt Ser-473 phosphorylation in HUVECs. A representative blot from three independent experiments is shown and the data expressed as mean ± SEM in each bar graph represent the average of three independent experiments. *P < 0.05 (Tun vs. control). ^#^P < 0.05 (Tun/ucOcn vs. Tun). IB, immunoblot; IP, immunoprecipitation.

Recently, it has been proved that insulin resistance in osteoblasts led to a decrease in circulating levels of the active form of osteocalcin^16^, suggesting that osteocalcin was linked with the development of insulin resistance. To shed light on the role of ucOCN and its downstream signaling pathway on insulin resistance in HUVECs, HUVECs were pretreated with 5 μg/ml tunicamycin (Tun) for 4 h to induce insulin resistance. Tun not only significantly increased protein expression of ATF4 and CHOP, and phosphorylations of PERK and eIF2α (Fig. 2A,B and Supplemental Figure 2A and B), but also decreased insulin-stimulated IRS-1 tyrosine phosphorylation and Akt Ser-473 phosphorylation (Fig. 2C and D). However, protein expression of ATF4 and CHOP, and phosphorylations of PERK and eIF2α were markedly reduced in HUVECs treated with Tun and ucOCN compared to HUVECs treated with Tun but not ucOCN (Fig. 2A,B and Supplemental Figure 2A and B). Simultaneously, IRS-1 tyrosine phosphorylation and Akt Ser-473 phosphorylation were significantly elevated (Fig. 2C and D), suggesting that ucOCN significantly alleviated Tun-induced ER stress and improved insulin signal transduction in the HUVECs. Furthermore, we also pretreated HUVECs with 500 μM palmitate to induce insulin resistance *in vitro*. As shown in Supplemental Fig. 1A–D, palmitate treatment induced ER stress and impaired insulin signaling in HUVECs, with such effects being reversed by ucOCN. Additionally, tunicamycin-induced HUVECs displayed reduced glucose uptake, and addition of uncarboxylated osteocalcin in tunicamycin-induced HUVECs increased glucose uptake (Supplemental Figure 2C).

### Effect of ucOCN on insulin signal transduction is mediated via PI3K/Akt/NF-κB signaling in HUVECs

**Figure 3:**
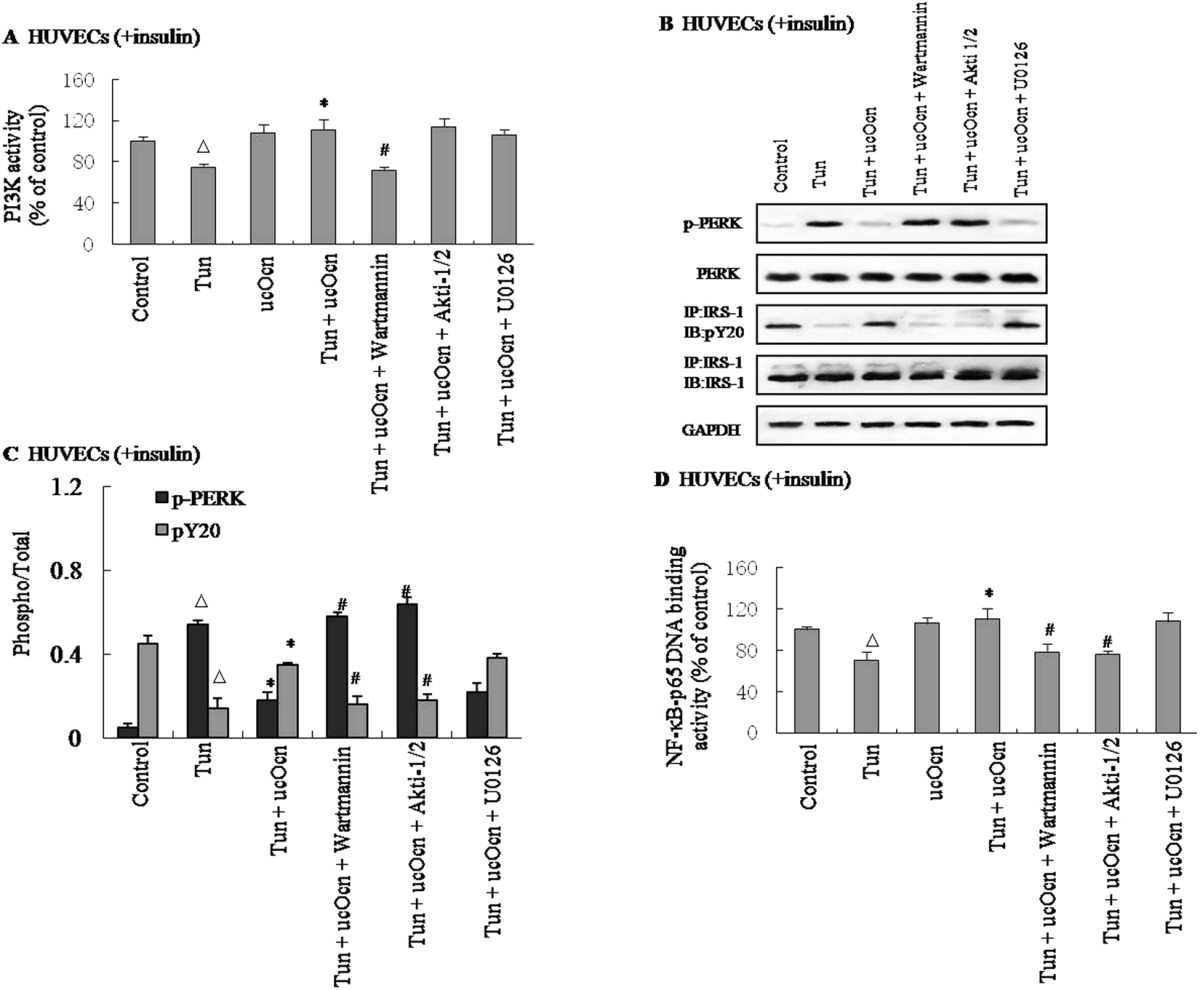
Effect of ucOCN on insulin signal transduction is mediated via PI3K/Akt/NF-κB signaling in HUVECs. Tunicamycin (Tun) was used to induce insulin resistance. For insulin signaling, cells were stimulated with 10 nM of insulin for 10 min. Cells were cultured in the presence or absence of uncarboxylated osteocalcin with or without specific signaling pathway inhibitors such as 10 μM wortmannin (a PI3K inhibitor), 10 μM Akti-1/2 (an AKT inhibitor) or 10 μM U0126 (a MAPK inhibitor) for 4 h. PI3K binding activity was determined by an *in vitro* kinase assay. NF-κB p65-DNA binding activity was determined by Elisa. The relative quantity of proteins was analyzed Quantity One software. (**A**) PI3K activity in HUVECs. (**B**) Phosphorylation of PERK and IRS-1 in HUVECs. (**C**) Densitometric analyses of PERK and IRS-1 in HUVECs. (**D**) NF-κB p65 DNA binding activity in HUVECs. A representative blot from three independent experiments is shown and the data expressed as mean ± SEM in each bar graph represent the average of three independent experiments. *P < 0.05 (Tun/ucOcn vs. Tun). ^#^ *P* < 0.05 (Tun/ucOcn/inhibitor vs. Tun/ucOcn). Δ*P* < 0.05 (Tun vs. control). IB, immunoblot; IP, immunoprecipitation.

The activation of NF-κB followed by Akt phosphorylation has recently been implicated in the development of insulin resistance^17^. Based on these results, we hypothesized that the intracellular PI3K/Akt/NF-κB signaling pathway is involved in the actions of ucOCN. To determine the underlying molecular mechanism, HUVECs were cultured in the presence of insulin, Tun, and/or ucOCN, with or without specific inhibitors of each signaling pathway such as wortmannin (a PI3K inhibitor), Akti-1/2 (an Akt inhibitor), and U0126 (a MAPK inhibitor). ucOCN significantly increased the PI3K activity in an *in vitro* kinase assay in HUVECs in the presence of insulin resistance (Fig. 3A). Meanwhile, the effects of ucOCN in improving ER stress and insulin signal transduction were nullified by the addition of wortmannin or Akti-1/2, but not by the addition of U0126 as analyzed by the phosphorylations of PERK and IRS-1 (Fig. 3B and C, Supplemental Figure 3A–D). In addition, NF-κB DNA-binding activity was measured using a NF-κB p65 transcription factor for ELISA. The addition of 10 μM wortmannin or Akti-1/2 significantly reversed the positive effects of ucOCN on NF-κB p65 DNA-binding activity, whereas the addition of U0126 did not (Fig. 3D).

**Figure 4:**
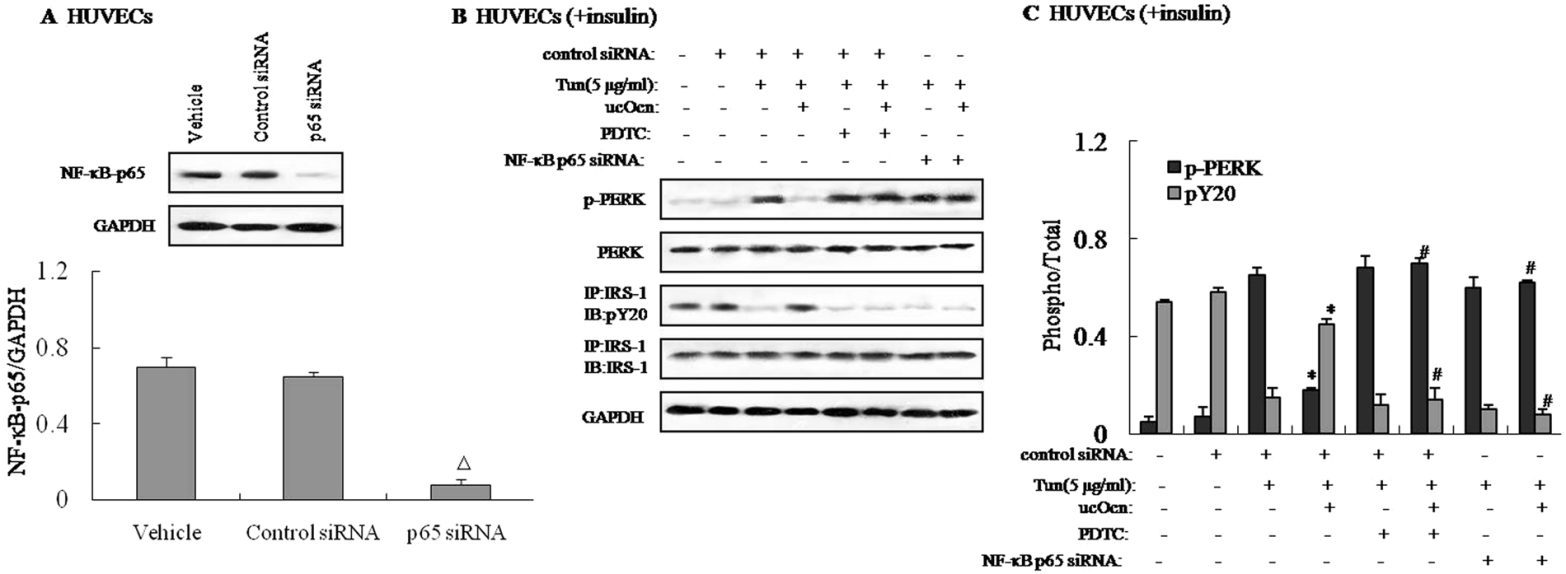
Effect of NF-κB blockade on ER stress and insulin signaling. Tunicamycin (Tun) was used to induce insulin resistance. HUVECs were cultured in the presence or absence of uncarboxylated osteocalcin with or without 1 μM PDTC (an NF-κB inhibitor) and 100 nM NF-κB-p65 siRNA. For insulin signaling, cells were stimulated with 10 nM of insulin for 10 min. The relative quantity of proteins was analyzed Quantity One software. (**A**) Protein expression of NF-κB-p65 in HUVECs. (**B**) Phosphorylation of PERK and IRS-1 in HUVECs. (**C**) Densitometric analyses of PERK and IRS-1 in HUVECs. A representative blot from three independent experiments is shown and the data expressed as mean ± SEM in each bar graph represent the average of three independent experiments. *P < 0.05 (Tun/ucOcn vs. Tun/Veh). ^#^ *P* < 0.05 (Tun/ucOcn/inhibitor vs. Tun/ucOcn). Δ*P* < 0.05 (p65 siRNA vs. control siRNA). IB, immunoblot; IP, immunoprecipitation.

To confirm that the NF-κB signaling pathway is causally involved in the regulation of ucOCN on ER stress and insulin signaling in HUVECs, this pathway was blocked by administering pyrrolidine dithiocarbamate (PDTC), a well-known NF-κB inhibitor, or NF-κB p65 siRNA. NF-κB p65 siRNA was validated by measuring the reduction in p65 protein expression by Western blotting in NF-κB p65-siRNA-transfected HUVECs (Fig. 4A). Additionally, blocking the NF-κB pathway by adding PDTC to the culture medium or by transfecting with NF-κB p65 siRNA in cells reversed the protective effects of ucOCN on Tun-induced ER stress and impaired insulin signaling (Fig. 4B and C). These findings indicated that ucOCN partially suppresses ER stress and improves insulin signal transduction via PI3K/Akt/NF-κB signaling in HUVECs.

## Discussion

OCN, as a synthetic osteoblast-specific protein, has been investigated in various research studies including fundamental animal studies and clinical trials, but its action mechanism remains elusive. ucOCN plays a key role in the development of insulin resistance. The present study has provided clinical evidence for a possible association of ucOCN with vascular complications of T2DM in Chinese men. Our results have also shown that ucOCN acts directly on HUVECs and partially suppresses ER stress and improves insulin signal transduction via PI3K/Akt/NF-κB signaling in Tun-induced HUVECs. Thus, ucOCN is linked to the vascular complications of T2DM, and it could potentially be used in treatments for the vascular complications of T2DM.

Some recent studies have shown that serum ucOCN and totOCN are related to T2DM in different racial groups^18, 19^. These studies found that reduced serum totOCN was associated with an increased risk of metabolic syndrome in older men, and that serum ucOCN was inversely correlated with adiposity, blood glucose, insulin resistance, and triglycerides. Consistent with these results, we found that the serum ucOCN and totOCN concentrations and the ucOCN/totOCN ratio were significantly lower in PDM and T2DM than in NGT in a Chinese population. Notably, our multiple logistic regression analysis showed that serum levels of ucOCN and totOCN were independent factors for the development of the vascular complications in this cohort. Moreover, univariate correlation analyses suggested the presence of negative correlations between ucOCN and WHR, FPG, FINS, HbA1c, and HOMA-IR, and a positive correlation between ucOCN and eGFR. Therefore, we speculated that OCN is a key protector of the development of the vascular complications of T2DM.

T2DM-related deaths are closely associated with the development of premature atherosclerosis and microvascular complications. Serum OCN levels have been found to be negatively associated with the brachial-ankle pulse wave velocity, intima-media thickness, and the risk of CAD^20, 21^. The present study also suggested an possible relationship between serum levels of ucOCN and the severity of the vascular complications of T2DM, revealing an association between serum OCN concentrations and CVD. However, the cross-sectional design of this study means that it could not address the cause–effect relationship of OCN with T2DM and CAD. Therefore, further large population-based prospective studies are warranted to confirm the independent predictive and protective role of OCN in the development of T2DM and CAD.

Additionally, an increasing number of evidence suggested a close link between osteoporosis and cardiovascular disease, because of common pathophysiological and genetic risk factors^22^. Actually vascular calcification not only is a passive process of calcium and phosphate absorption, but also is an active process, mediated by similar mechanisms in the osteogenesis process^23, 24^. It has been proved that the specific protein involved in bone mineralization such as bone morphogenetic protein and osteopontin could regulate vascular calcification^25^, suggesting that OCN as an osteoblast-specific protein might be associated with the progress of vascular complications of T2DM. Although a number of pathophysiological common features between osteoporosis and cardiovascular disease, molecular mechanisms still need to further explore *in vivo* and *in vitro*.

*In vivo* and *in vitro* studies have indicated that ucOCN exerts profound effects on glucose homeostasis and lipid metabolism. Consistent with these results, we also found that ucOCN may act on HUVECs directly and that ucOCN partially suppresses ER stress and improves insulin signal transduction via PI3K/Akt/NF-κB signaling in HUVECs. An unbiased approach based on the ability of OCN to increase cAMP production in Leydig cells and on its dichotomy of function between male and female gonads led to Gprc6a being identified as the OCN receptor^26^. Moreover, recent study also showed that Gprc6a was the OCN-sensing G protein-coupled receptor that directly regulates pancreatic β-cell functions^3^, but it is still difficult to clearly define the early stages of OCN-mediated signaling from the plasma membrane.

In summary, this study has provided clinical evidence that the serum level of ucOCN is decreased in subjects with vascular complications of T2DM, and that ucOCN is inversely associated with T2DM in Chinese men. Additionally, we found that ucOCN partially suppresses ER stress and improves insulin signal transduction via PI3K/Akt/NF-κB signaling in HUVECs, implicating ucOCN as a novel therapeutic target for the treatment of the vascular complications of T2DM.

## Electronic supplementary material

Supplemental Figure 1–3
