# Supplementary material for: Undercarboxylated osteocalcin reverts insulin resistance induced by endoplasmic reticulum stress in human umbilical vein endothelial cells
Source: Sci Rep. 2017 Mar 3;7:46. doi: 10.1038/s41598-017-00163-2 (PMC5427815; doi:10.1038/s41598-017-00163-2)

Undercarboxylated osteocalcin reverts insulin resistance induced by endoplasmic reticulum stress in human umbilical vein endothelial cells

Running title: Effects of ucOCN on insulin resistance

Qinyue Guo[1], Huixia Li[2], Lin Xu[3], Shufang Wu[4], Hongzhi Sun[2]*, Bo Zhou[1]*

[1]Department of Respiratory and Critical Care Medicine, the First Affiliated Hospital of Xi'an Jiaotong University, 277 Yanta West Road, Xi'an, Shaanxi 710061, China

[2]Key Laboratory of Environment and Genes Related to Diseases, Medical School of Xi'an Jiaotong University, Xi'an, Shaanxi 710061, China

[3]Department of Endocrinology, the Affiliated Guangren Hospital of Xi'an Jiaotong University, Xi'an, Shaanxi 710004, China

[4]Center for Translational Medicine, the First Affiliated Hospital of Xi'an Jiaotong University, 277 Yanta West Street, Xi'an, Shaanxi 710061, China

*Correspondence should be addressed to Bo Zhou: zb_bob@stu.xjtu.edu.cn. Department of Respiratory and Critical Care Medicine, the First Affiliated Hospital of Xi'an Jiaotong University, 277 Yanta West Road, Xi'an, Shaanxi 710061, China

*These authors contributed equally to this work.

Supplemental Figure 1. Effects of ucOCN on palmitate-induced ER stress and impaired insulin signaling in HUVECs. Palmitate (Palm) was used to induce insulin resistance in HUVECs. The relative quantity of proteins was analyzed using Quantity One software. (A) Protein expression of ATF4, CHOP and phosphorylation of PERK and eIF2α in HUVECs. (B) Densitometric analyses of ATF4, CHOP and phosphorylation of PERK and eIF2α in HUVECs. (C) IRS-1 tyrosine phosphorylation and Akt Ser-473 phosphorylation in HUVECs. (D) Densitometric analyses of IRS-1 tyrosine phosphorylation and Akt Ser-473 phosphorylation in HUVECs. A representative blot from three independent experiments is shown and the data expressed as mean ± SEM in each bar graph represent the average of three independent experiments. *P < 0.05 (Palm vs. control). $^{\#}$P < 0.05 (Palm/ucOcn vs. Palm). IB, immunoblot; IP, immunoprecipitation.

Supplemental Figure 2. (A) Protein expression of ATF4 and CHOP in HUVECs. (B) Densitometric analyses of ATF4 and CHOP in HUVECs. (C) Glucose uptake in HUVECs. *P < 0.05 (Tun vs. control). $^{\#}$P < 0.05 (Tun/ucOcn vs. Tun).

Supplemental Figure 3. (A) Phosphorylation of PERK and IRS-1 in HUVECs with or without Akti-1/2 in the presence or not of tunicamycin. (B) Densitometric analyses of PERK and IRS-1 in HUVECs with or without Akti-1/2 in the presence or not of tunicamycin. (C) Phosphorylation of PERK and IRS-1 in HUVECs with or without wortmannin in the presence or not of tunicamycin. (D) Densitometric analyses of PERK and IRS-1 in HUVECs with or without wortmannin in the presence or not of tunicamycin. *P < 0.05 (Tun/inhibitor vs. control).

Supplemental Figure 1

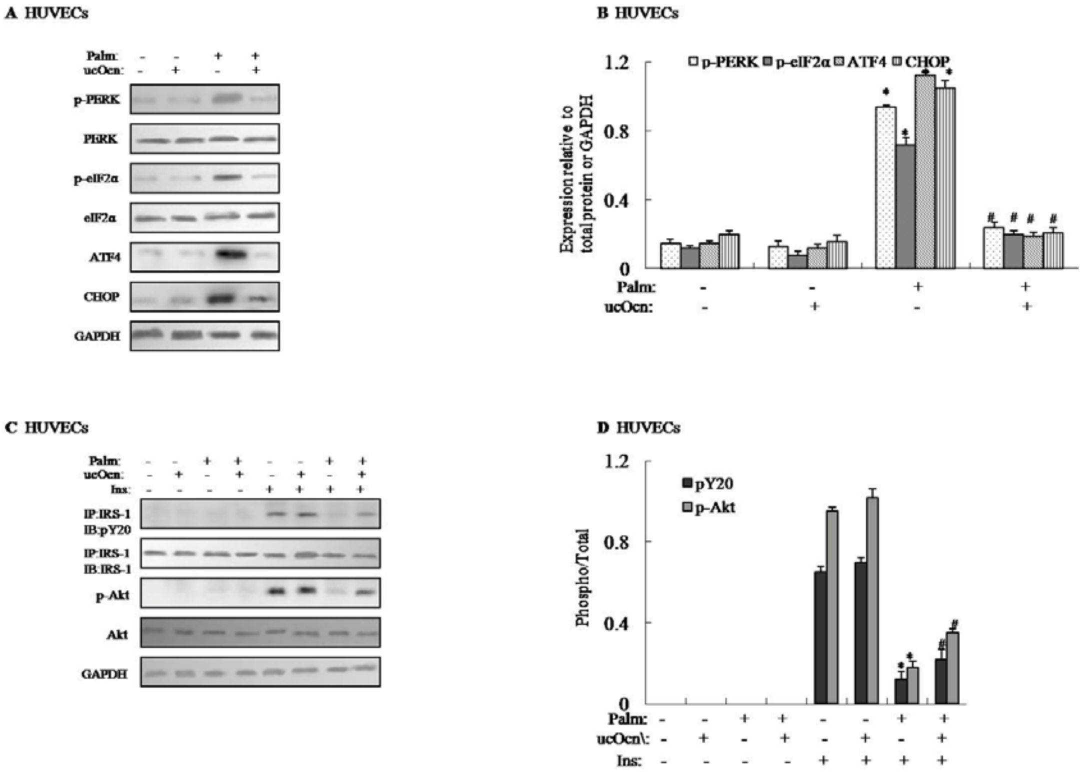

Supplemental Figure 2

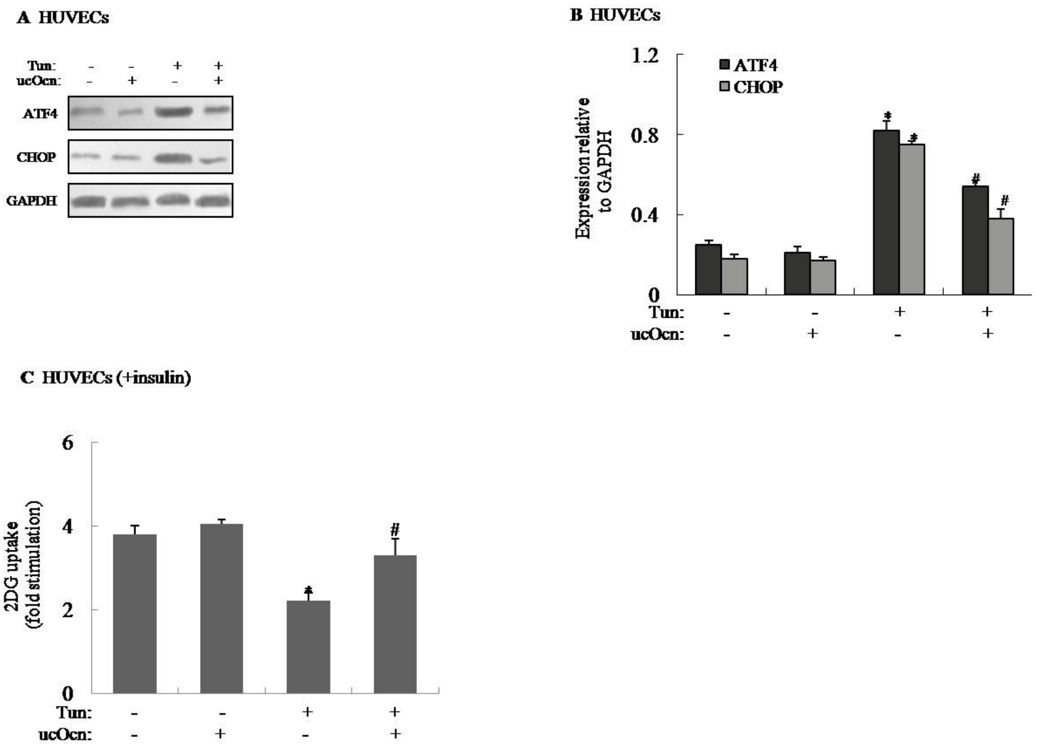

Supplemental Figure 3

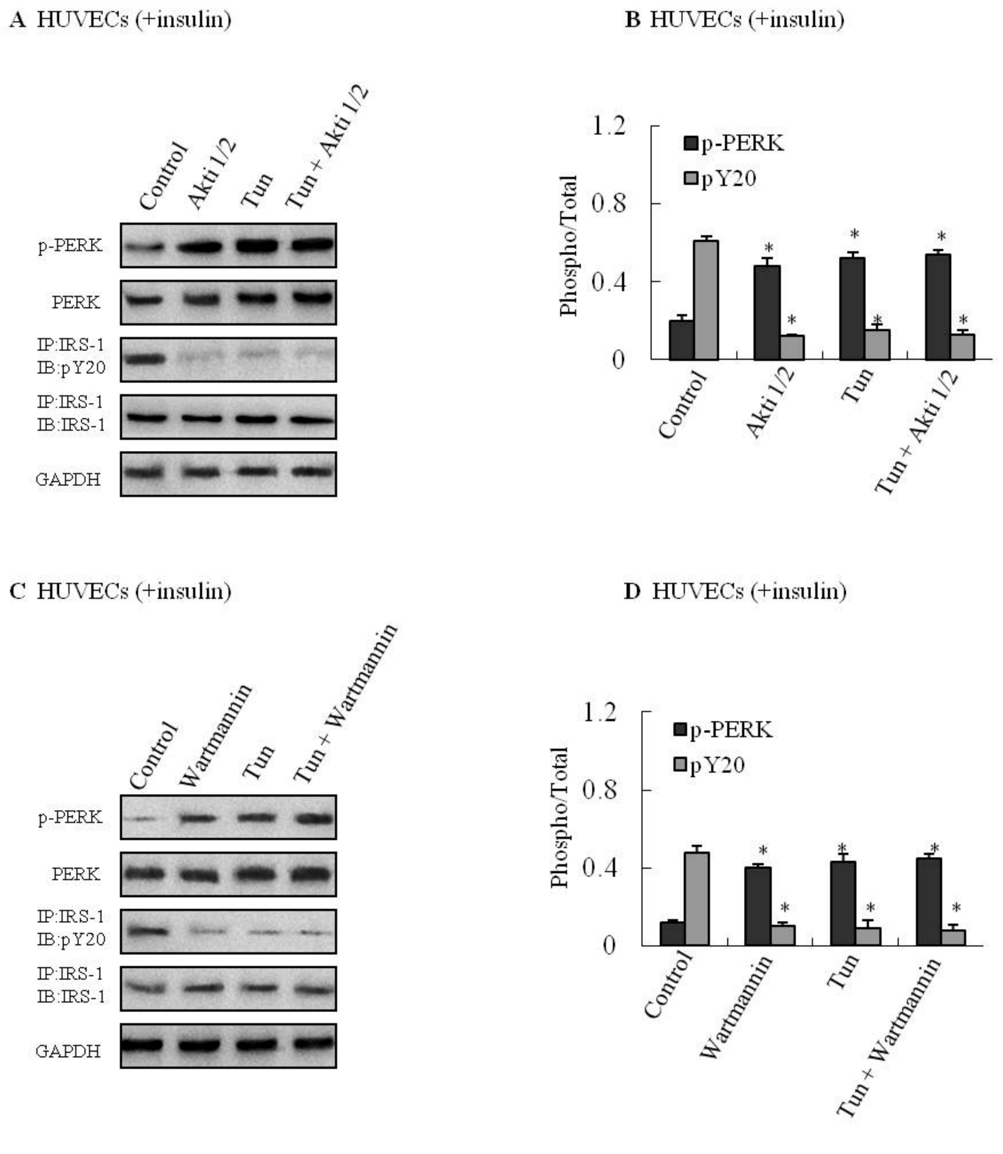

Supplement: Supplementary file 1 — Supplemental Figure 1–3 [file 41598_2017_163_MOESM1_ESM.pdf]
